# Haplotype-Based Genotyping in Polyploids

**DOI:** 10.3389/fpls.2018.00564

**Published:** 2018-04-26

**Authors:** Josh P. Clevenger, Walid Korani, Peggy Ozias-Akins, Scott Jackson

**Affiliations:** ^1^Mars-Wrigley Confectionery, Center for Applied Genetic Technologies, Athens, GA, United States; ^2^Institute of Plant Breeding, Genetics, and Genomics, College of Agricultural and Environmental Sciences, University of Georgia, Tifton, GA, United States; ^3^Institute of Plant Breeding, Genetics, and Genomics, University of Georgia, Athens, GA, United States

**Keywords:** polyploid SNPs, SNP array, *Arachis*, haplotype markers, sequence-based genotyping

## Abstract

Accurate identification of polymorphisms from sequence data is crucial to unlocking the potential of high throughput sequencing for genomics. Single nucleotide polymorphisms (SNPs) are difficult to accurately identify in polyploid crops due to the duplicative nature of polyploid genomes leading to low confidence in the true alignment of short reads. Implementing a haplotype-based method in contrasting subgenome-specific sequences leads to higher accuracy of SNP identification in polyploids. To test this method, a large-scale 48K SNP array (Axiom Arachis2) was developed for *Arachis hypogaea* (peanut), an allotetraploid, in which 1,674 haplotype-based SNPs were included. Results of the array show that 74% of the haplotype-based SNP markers could be validated, which is considerably higher than previous methods used for peanut. The haplotype method has been implemented in a standalone program, HAPLOSWEEP, which takes as input bam files and a vcf file and identifies haplotype-based markers. Haplotype discovery can be made within single reads or span paired reads, and can leverage long read technology by targeting any length of haplotype. Haplotype-based genotyping is applicable in all allopolyploid genomes and provides confidence in marker identification and in silico-based genotyping for polyploid genomics.

## Introduction

The identification of functional variation controlling traits of interest relies on the ability to discern all true variation between accessions with discrete genotypes. The power of next-generation (short reads) and third-generation (long reads) sequencing is the ability to identify all variants. The size and complexity of polyploid genomes have led to the reliance on single nucleotide polymorphism (SNP) arrays and complexity reduction sequencing strategies such as genotyping-by-sequencing (GBS) and restriction site-associated DNA sequencing (RADSeq; Elshire et al., 2011; Willing et al., 2011). These methodologies have allowed access to unprecedented number of markers for genomics. There are drawbacks in using these technologies, however. One is the inherent ascertainment bias in using SNP arrays for genotyping (Lachance and Tishkoff, 2013). Ascertainment bias occurs from the bias associated with sampling smaller populations. Since the SNP probes on arrays are static, rare variants or subpopulation-specific variants will not be assayed. This will cause bias in population genetics studies, and will not allow the identification of rare functional variants controlling traits of interest. A method of identifying markers straight from sequence data alleviates ascertainment bias on an experiment-wise level by providing access to all potential polymorphisms in the population of interest and does not constrain analysis to discrete markers on an array.

With the cost of sequencing continuing to plummet and long read technologies increasing in efficiency and accuracy, the ability to generate sequence on whole populations is increasing. Having access to all potential polymorphisms can increase the resolution of genetic mapping and genome-wide association studies. In polyploids, confidence in sequence-based prediction of genotypes is confounded by the uncertain alignment of short reads in the genome. Mapping of homeologous reads causes confusion by the appearance *in silico* of a polymorphism between accessions that is only between subgenomes. This problem is confounded by variance in sequence coverage across genomic loci.

A method of untangling subgenomes in mapping experiments has been proposed and implemented in cultivated peanut [sliding window extraction of explicit polymorphisms (SWEEP)] and octoploid strawberry (Bassil et al., 2015; Clevenger and Ozias-Akins, 2015). Bassil et al. (2015) did not report the accuracy of marker identification, but SWEEP performed well in simulations. SWEEP was used to design a large scale SNP array and showed that accuracy was useful although lower than estimated (Clevenger et al., 2017b). A more precise method is needed to assign subgenome specificity to mapped reads to more definitively identify polymorphisms between accessions.

We propose a method of sequence-based genotyping in polyploids that instead of applying a filter to individual sites collects observed haplotypes from sequence reads and contrasts those haplotypes between accessions to identify polymorphic markers. To demonstrate the accuracy of the method, haplotype-based markers were validated on a new 48k SNP array for *Arachis*, Axiom Arachis2. Finally, a pipeline was developed to utilize the haplotype-based genotyping method as an easy-to-use one command program. Haplotype-based genotyping should be broadly applicable across allopolyploid species.

## Materials and Methods

### Axiom Arachis2 Design

For SNP identification, a set of 21 *Arachis hypogaea* accessions were re-sequenced to 10X coverage (Clevenger et al., 2017b) and sequences from three accessions that are parents of two RIL populations [“T” (Qin et al., 2012) and “S” (Khera et al., 2016)] were also used. Analysis of sequence and SNP calling was carried out as in Clevenger et al. (2017b). Filtering for high-quality SNPs was then done using two methods. The first method, described here, was haplotype-based markers converted to SNPs. There were a total of 1,746 haplotype-based SNPs submitted to Affymetrix of which 1,674 were selected for the array. An alternative filtering method was used that took SWEEP-filtered SNPs (Clevenger and Ozias-Akins, 2015) and filtered them further using a machine learning approach (SNP-ML^1^). The models used for machine learning were trained using the true and false SNP sets from a previous array, Axiom Arachis v1 (Clevenger et al., 2017b; Pandey et al., 2017). A total of 133,162 putative SNPs were submitted to Affymetrix, of which 28,218 were selected for the array.

In addition, 6,407 markers between Tifrunner and GT-C20 were included that were identified using an early assembly of the cultivated Tifrunner genome ^2^. Potential SNPs were filtered by only taking those SNP sites where all Tifrunner reads contained the reference base and all GT-C20 reads contained the alternate base. The remaining 22 markers were added based on their utility in marker-assisted selection. Seven markers select for an alien introgressed region that controls nematode resistance on chromosome A09, including a marker that is within the current candidate gene for resistance (Clevenger et al., 2017a). Seven markers were selected for late and early leaf spot resistance identified using QTL-seq (Clevenger et al., 2018). Eight markers were selected for two alien introgressed regions from *Arachis cardenasii* that control late leaf spot and rust resistance (Pandey et al., 2016; Clevenger et al., 2017c). The final 11,516 markers were included from the Axiom Arachis v1 that were identified as useful in interspecific populations. Of these, 4,489 were high-quality polymorphic markers in *A. hypogaea* populations. Supplementary Table S1 provides the final design of the Axiom Arachis2 SNP array.

### Haplotyping Workflow

To identify haplotype-based markers, all possible polymorphic sites were called using Samtools mpileup. These potential polymorphisms were then used as a guide for the haplotyping procedure. The program is written in C++ as a standalone program. To access bam files and retrieve reads aligned to specific locations, the bamtools API is used (Barnett et al., 2011). First, all haplotypes for each accession are collected at every two-position haplotype where there is a potential polymorphism at both sites within a specified base window. Haplotypes are only collected if they occur within a single contiguous read. The haplotypes are stored in a data frame that is organized by genotype, haplotype position, and counts for each observed haplotype. Then the stored haplotypes are filtered based on the following criteria: (1) for a given pair of SNPs, there must be at least two accessions with more than one haplotype; (2) within each of these two accessions, both of its haplotypes must be observed at least twice; (3) within each of these two accessions, reads supporting the least observed haplotype must be at least 25% as frequent as the reads supporting the most observed haplotype (to exclude rare haplotypes that could be due to sequence error in one of the accessions); and (4) in at least one, but not all, accessions, the two haplotypes must be the same for one site and different from each other at the other site.

There are three different run modes to identify haplotypes. The default mode is described as above as haplotypes are collected only within a single contiguous read. Additionally, paired-end information can be leveraged as haplotypes present within two pairs will be considered. This mode is most useful when using large insert size libraries as haplotypes can be considered that span longer distances. The third mode is a simple “diploid” mode where an additional parameter to adjust the number of bases considered for each haplotype is included. For this mode, the user can specify haplotypes from two up to *N* bases in length. This mode is useful when using long read technology as high-resolution haplotypes can be assayed.

The haplotyping procedure can be called individually or as a part of a pipeline. The pipeline can additionally take called haplotypes and genotype a population of individuals at those sites, giving as output an m × n matrix of genotypes, where m is individuals and n is haplotype markers. Usage and help file information is provided as a README in Supplementary File S1. HaploSWEEP is available under the MIT license at https://github.com/jclev-uga/HAPLOSWEEP.

### Array-Based Marker Validation

Array genotype calls were manually curated within the Axiom Analysis Suite 3.1^3^ based on methods described in Clevenger et al. (2017b). Each genotype that was used to design the array markers using HAPLOSWEEP was assayed with the array in duplicate. For validation of haplotype-derived markers, there were two considerations: (1) the marker shows true polymorphism between genotypes and (2) the genotype calls on the array match those called from the sequence data.

## Results and Discussion

### Haplotype-Based Genotyping Identifies High-Quality Polymorphic SNPs in Polyploids

In an allopolyploid genome, there are generally at least two copies of any chromosomal region. As the divergence of the subgenomes decreases, the co-linearity and sequence similarity increase. When utilizing sequence-based genotyping, short reads originating from each subgenome can both map to the same duplicate location. Because of variance in coverage between sequenced samples, the reads do not always map in the ratio expected, i.e., 50% for each in an allotetraploid, 33% in an allohexaploid, etc.

The question becomes how to differentiate *in silico* between reads originating from each subgenome? One proposal is that using expected sequence coverage and eliminating from consideration any region with higher than expected coverage will properly filter out the regions where both subgenomes map simultaneously. Unfortunately, this strategy does not protect against false positive SNP calls. As an example in peanut, consider three samples sequenced using whole genome shotgun (WGS) sequencing with an expected sequence coverage of 10X. A coverage-based strategy would suggest that any sites be ignored with coverage above 15 reads and below four reads for each accession. Using the diploid progenitor genome sequences, an estimate can be made of the number and location of polymorphisms between subgenomes by fragmenting one genome into overlapping short sequences and mapping them to the alternate genome. Doing this with *Arachis duranensis* (A genome) fragments mapped to *Arachis ipaensis* identifies potentially 8,605,615 polymorphic sites that represent potential false positive SNP calls. After calling SNPs between the three accessions and filtering for expected coverage, there are 2,898,744 of those sites that fall within the expected coverage. In an experiment using coverage-based filtering, all these SNPs are potential false positives. Further, there are 25,459 sites where at least one of the three genotypes is scored as “homozygous” which would be considered a true SNP. Looking at each accession separately and filtering for expected coverage, there are 213,791, 113,340, and 81,120 false SNP sites where only one allele is represented and would be called SNPs, but are false positives. Given that the potential true polymorphisms between accessions are low, these potential false positives would drown out the true signal in a sequence-based genotyping experiment. Even when filtering for higher than expected coverage, the potential is high that many of the SNPs identified will be false positives.

Recently, a pipeline was developed called SWEEP (Clevenger and Ozias-Akins, 2015). This approach differentiates between subgenomes using an “anchor” within a window upstream and/or downstream of a potential SNP site. The anchor is simply an identified polymorphism between subgenomes. Using the genotypic likelihoods calculated from samtools mpileup (Li et al., 2009), putative true positive SNPs are selected when an anchor is within the base pair window in all of the accessions considered and at the SNP site at least one accession has a homozygous call. In simulations, SWEEP selected true SNPs with a rate above 95%; however, using real data, the validation rate was much lower, as seen after design of a 58K SNP array (Clevenger et al., 2017b; Pandey et al., 2017). The limiting factor is still being able to accurately differentiate the two subgenomes correctly using short reads.

**FIGURE 1:**
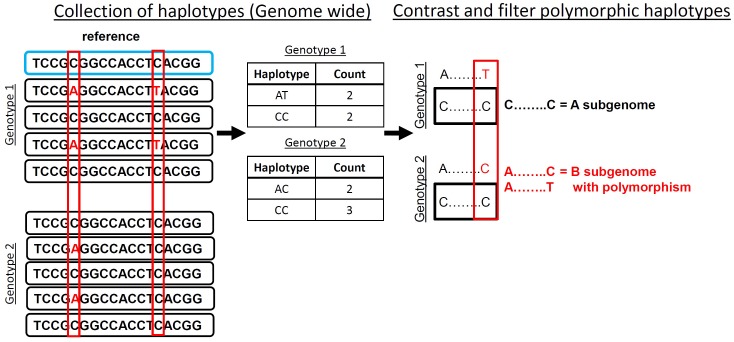
HaploSWEEP pipeline. Top sequence (blue box) represents the reference being aligned too with reads (black boxes) representing two genotypes aligned to that sequence. All haplotypes (in polyploid mode all two base haplotypes; in diploid mode, the user can specify the length of the haplotype) are collected genome-wide that are within the user-specified read length and contain at least one potential polymorphism. Then the haplotypes are analyzed for potential haplotype polymorphisms that can be distinguished from differences between subgenomes. The filtering process, outlined in Section “Materials and Methods,” is implemented and high-quality polymorphic haplotypes are output in two different files. One file lists every observed haplotype with number of observations in each accession. The other file lists the polymorphic haplotypes for each accession.

Accurate identification of polymorphisms from sequence data in polyploids can be done after contrasting collected observed haplotypes within contiguous sequence reads that align to a common locus. A polymorphic haplotype is identified by containing at one position an “anchor” base that is the same in all observed haplotypes and a polymorphic position that is observed in at least one accession (**Figure 1**). To demonstrate this method, we developed HAPLOSWEEP, which collects all observed overlapping haplotypes within a user-specified base pair window and contrasts them between accessions to identify those haplotypes that are polymorphic between accessions (**Figure 1**).

### Validation of the Haplotyping Method Using a 48K SNP Array

A 48K SNP array was developed for *Arachis*, Axiom Arachis2. A total of 1,674 haplotype-based markers were included on this array. To validate these haplotype-based markers, the parents used to identify them *in silico* were assayed on the array in duplicate. Analysis revealed that 1,243 (74%) of the haplotype-based markers could be validated (Supplementary Table S2). Of the 431 that were not validated, further analysis of their probe sequences revealed that the sequences were highly repetitive. The average positions aligned to completely with greater than 94% identity in validated marker probes was 1.9 where for invalidated marker probes it was 5.7. The enrichment of repetitive probes that are false positives will not allow a clear segregation of the polymorphic locus and so it is unclear if the *in silico* identified haplotype marker is a false positive or not.

Even with a high-quality polyploid reference genome, SNP calling is not trivial. Of the 6,407 markers that were identified by mapping to an early tetraploid assembly, only 2,888 (45%) were validated on the array. This result confirms that the problem of the multiple mapping of short reads is not fully solved by using a polyploid reference assembly.

The remaining markers were identified using a machine learning approach outlined in Korani et al. (unpublished). These markers were validated with a true positive rate (TPR) of 75%.

The true and false SNP sites obtained from both SNP arrays provide a resource for estimating validation rates. The sets provide 44,087 validated true SNP sites and 42,767 validated false SNP sites. These sets can be used to estimate validation for called haplotypes. Using whole genome resequencing from accessions used to design the array, haplotype markers were called in sets of two, three, four, and five accessions (Supplementary Table S3). The called haplotype markers were then compared to the true and false sets from the arrays for overlapping sites. Given the ratio of true to false positive known sites, the expected overlap of called markers is significantly greater with the true set and significantly lower with the false set for all experiments. The average estimated TPR across all experiments is greater than 89%. These validation results combined with the array-based validation show that the haplotype-based genotyping method can produce reliable genotyping results straight from sequence data.

### Probability of Identifying Markers Genome-Wide

A caveat to the haplotype-based genotyping method described here is that a polymorphism must be in proximity to an anchor (subgenome polymorphism) within a distance that can be observed on a contiguous short read. In peanut, there are potentially 8,605,615 polymorphisms between the A and B subgenomes. These polymorphisms were identified by mapping fragmented, overlapping sequences from *A. duranensis* to the *A. ipaensis* genome. Given an experiment using 150 bp reads, there are 2,581,684,500 base pairs that are potentially within the range of those polymorphisms. With an estimated genome size of 2.7 billion base pairs, there is the potential to identify the majority of polymorphisms between any set of peanut accessions given a sequencing depth of at least 10-fold genome coverage.

Increased sensitivity and utility can be accomplished by incorporating paired read information. With that aim, a separate module was designed called HAPLOSWEEP_LONGRANGE. This module uses the same methodology to contrast observed haplotypes between accessions, but collects them differently. The data structure stores information on every read’s status as a pair and the aligned base at every queried position. After haplotypes are captured that occur within single reads, an additional step is done to collect all haplotypes that occur between paired reads. Then the paired haplotypes are contrasted in the same way as haplotypes occurring within single reads. Utility for this module is that large insert size libraries can be used to identify polymorphic markers across long distances.

### Long Read Utility and Utility in Diploid Genomes

The haplotype-based genotyping framework can be applicable to diploid crops as well. Using long read technologies or large insert size libraries, long haplotypes can increase the resolution of genotypes and precision of mapping. An additional module, HAPLOSWEEP_DIPLOID, was designed to contrast haplotypes of any length. As haplotypes are observed, the program collects all haplotypes within the user-specified window up to the maximum haplotype length specified. When the window is not large enough to observe a maximum length haplotype, shorter haplotypes are collected.

### Full Functionality

The modules described above are a part of a one-command pipeline that takes as input a vcf file of called SNPs and bam files of alignments. Maximum utility is achieved if no filtering of the vcf file is done beforehand as the haplotyping method needs to have access to all possible polymorphisms. The user can call any of the three modules based on needs of the experiment and set window size (length of reads) and max haplotype length. Additionally, given a set of called haplotypes, a population of individuals can be genotyped with the output a matrix of called genotypes for each individual.

### Flexibility

Cultivated peanut is an allotetraploid. It is the simplest case to illustrate the method described because there are only two subgenomes. Further, the progenitor genomes have been sequenced and can be utilized with this method. Peanut is a special case because the progenitor genomes are very similar to the cultivated genome (Bertioli et al., 2016). In fact, the *A. ipaensis* genome is estimated to show 99.96% similarity to the cultivated B subgenome (Bertioli et al., 2016). In this case, the diploid genomes of peanut act as a proxy for the cultivated reference genome. The functionality of the pipeline is suitable for a case such as this as well as a polyploid reference genome.

As far as higher order allopolyploids such as wheat (hexaploid) or strawberry (octoploid), the pipeline should be applicable. It is designed to find at least one contrasting haplotype. As long as the haplotypes are the same on the other subgenomes, the markers will be identified. In a situation where there are more than two alleles at a specific site, these sites may be filtered out in a higher order polyploid. The pipeline is not designed to work for autopolyploids.

## Conclusion

Large-scale validation using the Axiom Arachis2 48k SNP array has shown that haplotype-based genotyping in polyploids is more accurate than other methods. By leveraging the true and false SNP sites derived from the version 1 and version 2 *Arachis* SNP arrays, estimates of TPRs are above 89% for novel marker discovery. In order to allow all users access to this method, we developed a one-command pipeline that will call haplotype-based markers and use them to genotype populations. Shown to be accurate in allotetraploid peanut, this method should provide accurate marker identification in all allopolyploid species.

## Author Contributions

JC, PO-A, and SJ conceptualized the research. JC and WK performed the experiments, conducted data analysis, and curated data. JC wrote the original draft and was responsible for data visualization. SJ, PO-A, and JC revised the manuscript.

## Conflict of Interest Statement

The authors declare that the research was conducted in the absence of any commercial or financial relationships that could be construed as a potential conflict of interest.

## Footnotes

1 https://github.com/w-korani/SNP-ML/wiki

2 peanutbase.org

3 www.thermofisher.com
